# Editorial: Case reports in PET imaging 2024

**DOI:** 10.3389/fmed.2026.1884774

**Published:** 2026-06-02

**Authors:** Carmelo Caldarella, Matteo Bauckneht, Romina Grazia Giancipoli

**Affiliations:** 1 Nuclear Medicine Unit, Dipartimento di Diagnostica per Immagini e Radioterapia Oncologica, Fondazione Policlinico Universitario Agostino Gemelli IRCCS, Rome, Italy; 2 IRCCS Azienda Ospedaliera Metropolitana, Genova, Italy; 3 Department of Health Sciences (DISSAL), University of Genova, Genova, Italy; 4 PET-CT Center, Fondazione Policlinico Universitario Agostino Gemelli IRCCS, Rome, Italy

**Keywords:** ^18^F-FDG, ^68^Ga-DOTATATE, ^68^Ga-PSMA, case report, diagnostic performances, PET-CT imaging, uncommon disease

Over the years, the role of hybrid positron emission tomography-computed tomography (PET-CT) imaging has become increasingly established in the study of a wide range of pathophysiological processes—particularly in oncology field—thanks to the growing number of radiopharmaceuticals able to detect specific molecular targets with increasing accuracy.

The Research Topic “*Case reports in PET imaging 2024*” includes 11 papers, specifically one case series and 10 case reports, concerning rare neoplasms, atypical disease locations, or potentially confusing/challenging benign conditions in which the role of functional information provided by PET-CT imaging has been crucial in staging and identifying unexpected disease sites, differential diagnosis, selecting the biopsy site, and evaluating response to treatment. These articles demonstrate that PET-CT is particularly useful when conventional morphological imaging is ambiguous, the site of disease is uncommon, or when distinguishing between a primary lesion, metastasis, recurrence, or a benign condition mimicking a neoplasm is necessary. However, we need to be aware that metabolic and receptor imaging must always be interpreted in an integrated, context-specific manner, taking into account the clinical history, anatomical location, uptake pattern, morphological imaging, and histological examination. In this context, PET-CT plays a complementary role to other imaging modalities, not only improving staging but also modifying management, guiding biopsies, and documenting early therapeutic response or disease recurrence.

The case series by Zhou, Li et al. has examined the role of ^18^F-FDG PET-CT in the management of plasmacytoid dendritic cell leukemia, a rare and aggressive hematological malignancy. The disease primarily affects the skin (89% of cases), lymph nodes (39%), and bone marrow (62%) (1). ^18^F-FDG PET-CT revealed hypermetabolic lesions even in uncommon extra-nodal sites such as lungs and breasts. The study of 18 cases showed increased ^18^F-FDG uptake in all the patients (SUVmax mean 9.1; range 1.5–9.1). Isolated cutaneous lesions showed moderate-to-high SUVmax (2.8–6.7); multiple cutaneous lesions exhibited milder uptake (1.5–2.6); lymph nodes showed moderate to high uptake, ranging from 2.3 to 6.8. ^18^F-FDG PET-CT proved to be useful for initial staging, identification of biopsy site, and response assessment. Furthermore, such data suggest that SUVmax may have a prognostic value, since patients with higher SUVmax exhibit a more rapid and unfavorable outcome: the analysis indicated that an initial SUVmax > 2.5 was associated with shorter survival (death within 2 months from diagnosis), whereas patients with SUVmax < 2.5 had much longer survival (up to 34 months).

A common theme across all the articles included in this Research Topic is the crucial role of PET-CT imaging in identifying uncommon disease sites that are not suspected at disease onset or are difficult to interpret on conventional morphological imaging. In cases of rare metastases, such as widespread pancreatic involvement from paranasal sinus rhabdomyosarcoma (Hu, Duan et al.) or metastasis to the small intestine from a large-cell lung cancer (Jiang et al.), PET-CT helped directing the diagnostic suspicion toward the possible presence of a metastasis, which was subsequently confirmed histologically. In these scenarios, the presence of new symptoms, such as abdominal pain or gastrointestinal disturbances, in previously treated cancer patients should prompt consideration of an uncommon metastatic site as differential diagnosis, in the clinical management. Thus, PET-CT has been useful not only in identifying rare metastases and distinguishing between local recurrence and distant disease, but also in the early detection of particularly uncommon intestinal metastases—particularly small or submucosal lesions not identifiable on morphological imaging—in patients with previous history of cancer and new onset of abdominal symptoms, even several years after the treatment of the primary tumor.

Similarly, in rare extra-nodal lymphomas, such as primary diffuse large B-cell lymphoma of the bladder (PB-DLBCL) (Li et al.), ^18^F-FDG PET-CT may play a significant role in determining the extent of the disease. However, the baseline ^18^F-FDG PET-CT was limited by high physiological radiourinary activity: to overcome these limitations, intravenous furosemide was administered, and delayed imaging was performed 2 h after ^18^F-FDG administration (1 h after the diuretic). The measures adopted reduced background activity in the bladder and enabled visualization of the metabolically active bladder wall lesion, thereby providing more accurate staging. This case demonstrates the need to adapt the acquisition protocol to the anatomical site. Physiological radiourinary activity may conceal metabolically active bladder wall lesions; therefore, using furosemide and obtaining delayed imaging could improve lesion visualization and allow for an accurate staging. After four cycles of R-CHOP, post-treatment ^18^F-FDG PET.CT showed a complete metabolic response. In literature, dual-phase ^18^F-FDG PET-CT with furosemide administration has been used in several cases for staging, follow-up, and response assessment, showing a reduction or disappearance of hypermetabolic lesions (2–5). Furthermore, higher SUVmax values were associated with a poorer prognosis, although the limited number of cases precludes definitive conclusions.

Another case report of this Research Topic described a rare case of intestinal NK/T-cell lymphoma that can present with non-specific abdominal symptoms and malignant peritoneal effusion (Zhou, Zhao et al.). The patient presented with abdominal distension, weight loss, ascites, and localized thickening of the small intestine. ^18^F-FDG PET-CT, cytology, and immunohistochemistry allowed the diagnosis. Despite chemotherapy, the course of the disease was rapidly unfavorable, with death shortly occurring after only 4 months. The ^18^F-FDG PET-CT was crucial for ruling out other primary sites and assessing the extent of the disease.

Regarding bone disease, two case reports have described rare and aggressive sites, such as a primary malignant giant cell tumor of the sacrum (Feng et al.) and a solitary plasmacytoma of the tibia (Hu, Li et al.) identified by ^18^F-FDG PET-CT, finally confirmed by histological examination.

Malignant giant cell tumor of bone (MGCTB) is extremely rare, accounting for approximately 1.6% of giant cell tumors of bone (GCTB) (6). It is classified as primary (PMGCTB) if initially diagnosed with sarcomatous components, and secondary if resulting from post-treatment malignant transformation (7). In this case report, a sacral PMGCTB developed in a 46-year-old patient with no prior medical history and lumbosacral pain for 4 months. An ^18^F-FDG PET-CT scan revealed high uptake in the sacrum, with SUVmax of 33.6, markedly higher than the average for benign GCTB tumors (approximately 9.2–10.4). ^18^F-FDG PET-CT was decisive in distinguishing the MGCTB from other spinal lesions, such as conventional GCTB, which is characterized by increased metabolism but significantly lower (8); the chordoma, which primarily affects the sacrococcygeal region, with a mean pre-treatment SUVmax of 5.1 (9), and bone metastases, which are usually associated with known history of a primary tumor and involvement of the vertebral pedicles (10). The intense ^18^F-FDG uptake in the sacral lesion suggested a malignant biological behavior, specifically a malignant spinal tumor. The diagnosis was confirmed histologically, with high-grade sarcomatous components identified. The patient received denosumab, but the response was poor, and the disease progressed rapidly.

Solitary plasmacytoma is a localized clonal proliferation of neoplastic plasma cells, accounting for less than 5% of all multiple myelomas (11). Hu, Li et al. reported a rare case of recurrence of a solitary tibial plasmacytoma following surgery. An ^18^F-FDG PET-CT examination revealed intense uptake in the surgical area of the original proximal tibial lesion, corresponding to a soft-tissue mass at the co-registered CT scan, confirming the suspicion of local disease recurrence.

Liu and Zhao described an extremely uncommon case of hepatocellular adenocarcinoma (HAC) of the cervix found in a post-menopausal woman with elevated serum alpha-fetoprotein. It is an extra-hepatic neoplasm with hepatocellular differentiation, often associated with elevated alpha-fetoprotein levels, which primarily affects post-menopausal women and presents with irregular vaginal bleeding (12). ^18^F-FDG PET-CT images showed increased glucose metabolism, and patchy foci of increased FDG uptake were seen in the uterine cavity, which, combined with the clinical and pathological examination, suggested the possibility of endometrial hepatic-like adenocarcinoma, confirmed by the post-operative pathological sections.

Some papers from this Research Topic highlighted the usefulness of radiopharmaceuticals other than ^18^F-FDG for differential diagnosis, keeping in mind that metabolic or receptor imaging does not always imply malignancy. A relevant example is the case report by Hu, Zhao et al. describing a rare adrenal splenosis diagnosed in a patient with a medical history of pancreatic tail resection and splenectomy 8 years ago due to a neuroendocrine tumor in the pancreas tail with the spleen invasion. The adrenal lesion showed mild ^18^F-FDG uptake and intense ^68^Ga-DOTATATE uptake, a pattern potentially suggestive of metastasis from a neuroendocrine neoplasm, particularly in patients with a history of splenectomy and cancer. Histological examination revealed ectopic splenic tissue. Ectopic splenic tissue may show intense uptake of radiolabeled somatostatin analogs due to the expression of somatostatin receptors in splenic immune cells and vascular structures (13). Therefore, splenosis can represent a significant interpretive pitfall in PET-CT scans using radiolabeled somatostatin analogs. In splenectomised patients, an abdominal or adrenal lesion should also prompt consideration of this differential diagnosis on ^68^Ga-DOTATATE PET-CT.

Another case report described a rare pituitary metastasis from prostate cancer characterized by intense focal uptake on ^68^Ga-PSMA PET/CT in a patient with a medical history of prostate adenocarcinoma (Bianchetto Wolf et al.). When conducting a differential diagnosis of sellar lesions, pituitary adenomas or other benign lesions should also be considered. The diagnosis was confirmed by histology, immunohistochemistry, and methylation profiling, which demonstrated the prostatic origin of the pituitary lesion. The patient subsequently responded well to systemic therapy consisting of androgen deprivation, abiraterone, and docetaxel.

Finally, the assessment of therapy response could be improved by next-generation PET-CT scanners, such as those equipped with a long axial field of view (LAFOV), which are approximately ten times more sensitive than traditional digital systems, and allow to obtain a whole-body PET-CT examination in a really short time (2–5 min). Abgral et al. reported a complete metabolic response lasting more than 23 months in a patient with poorly differentiated metastatic pancreatic adenocarcinoma treated with FOLFIRINOX chemotherapy. The complete metabolic response was assessed using LAFOV ^18^F-FDG PET-CT and was maintained for over 23 months, concurrent with normalization of tumor marker CA19-9 levels. The authors emphasized that complete responses in metastatic pancreatic cancer are exceptional and that the greater sensitivity of LAFOV systems may enhance the assessment of metabolic response. The authors noted that next-generation ^18^F-FDG PET-CT, using a more sensitive LAFOV system, has an excellent negative predictive value, highlighting its potential for therapeutic assessment of solid cancers in future clinical trials.
